# Supplementary material for: Classification and spatiotemporal correlation of dominant fluctuations in complex dynamical systems
Source: PNAS Nexus. 2025 Feb 7;4(2):pgaf038. doi: 10.1093/pnasnexus/pgaf038 (PMC11833705; doi:10.1093/pnasnexus/pgaf038)
Supplement: pgaf038_Supplementary_Data [file pgaf038_supplementary_data.zip › PNASNEXUS-PNASNEXUS-2025-00078-T-s01.pdf]

PNAS nexus

# **Supplementary Information for**
Classification and Spatiotemporal Correlation of Dominant Fluctuations in Complex Dynamical Systems

Cristina Caruso, Martina Crippa, Annalisa Cardellini, Matteo Cioni, Mattia Perrone, Massimo Delle Piane, and Giovanni M. Pavan

Giovanni M. Pavan
Email: giovanni.pavan@polito.it

**This PDF file includes:**

Figs. S1 to S9
Table S1
Legends for Movies S1 to S2
SI References

**Other supplementary materials for this manuscript include the following:**

Movies S1 to S2

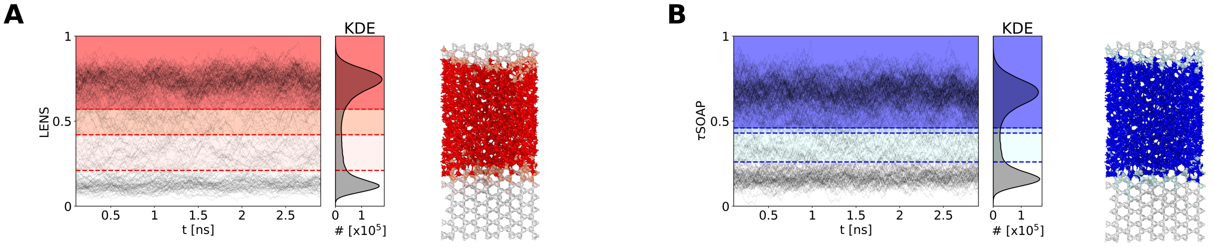


**Fig. S1. Univariate Onion clustering([1]) analysis on LENS and $\tau$SOAP time-series in Ice/liquid water phase coexistence.** LENS (A) and $\tau$SOAP (B) time-series, with the corresponding Kernel Density Estimation (KDE) distributions, are computed, for each oxygen (OW), in a system containing 2048 **TIP4P/Ice**([2]) water molecules (half in the crystalline hexagonal ice phase, half in the liquid phase) and simulated at T=267.5 K, namely at the solid/liquid transition temperature for the employed model.([2, 3]) The analysis is related to 3 ns extracted from the last part of a 100 ns-long MD simulation, sampled every $\Delta$t=0.001 ns. Both LENS and $\tau$SOAP signals are smoothed using a moving average with width=0.2 ns (200 frames). Onion clustering is applied using a time resolution of 0.25 ns (250 frames). (A) Left: Univariate Onion clustering applied on the LENS time-series. Four clusters are detected (LENS thresholds=[0.21, 0.42, 0.57]), colored from white to red. Right: Molecular Dynamics (MD) snapshots colored according to the detected clusters on the left, using the same color code. Four distinct domains are captured: Ice (white), solid/liquid interfaces (mistyrose and lightsalmon, respectively), and liquid water (red). (B) Left: Univariate Onion clustering applied on the $\tau$SOAP time-series. Four clusters are captured ($\tau$SOAP thresholds=[0.26, 0.43, 0.46]) and colored from white to blue. Right: MD snapshot, colored according to the detected clusters, is shown. Four distinct domains can be observed: Ice (white), solid/liquid interfaces (lightcyan and lightblue, respectively), and liquid water (blue). Both snapshots are taken at t$\sim$1.2 ns.

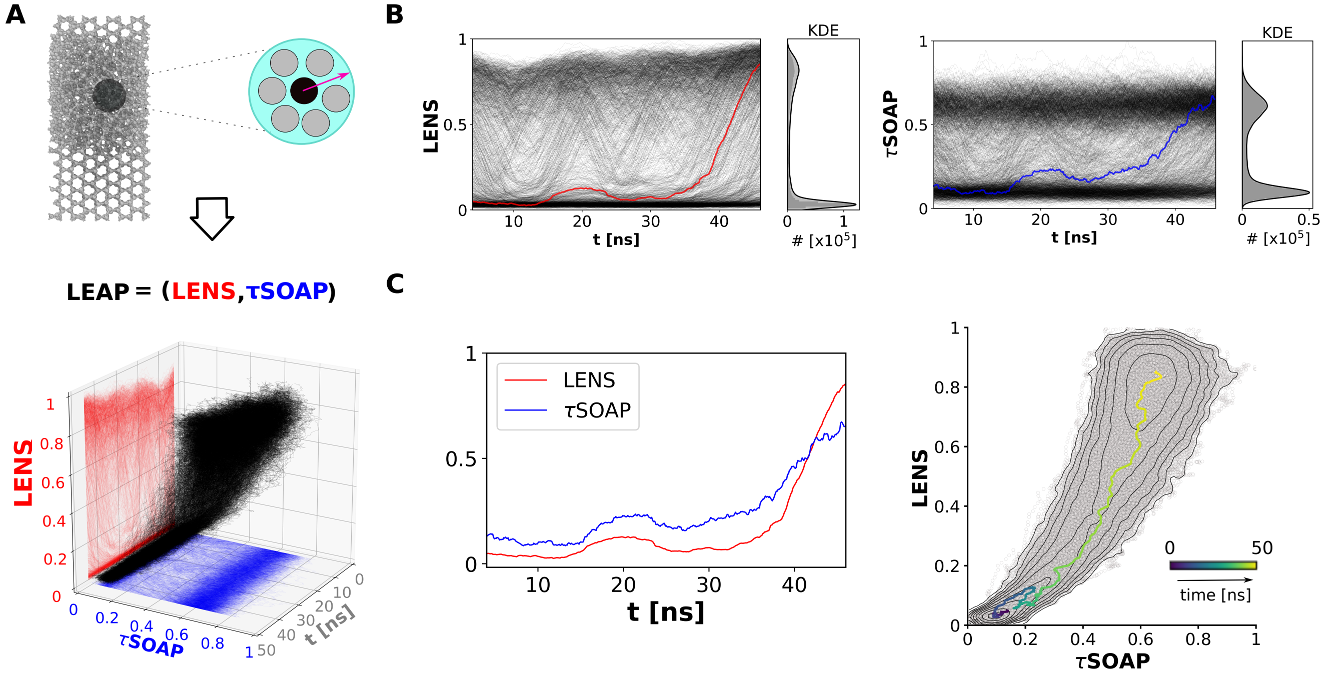


**Fig. S2. LEAP analysis in Ice/liquid water phase coexistence extended to 50 ns of a 100 ns-long MD trajectory.** (A) **LEAP** time-series dataset related to the whole second half of a 100 ns-long MD trajectory (sampling time $\Delta$t = 0.1 ns) composed of 2048 water molecules (**TIP4P/Ice** water model([2])), whose 50% in the crystalline hexagonal ice configuration and the remaining 50% in the liquid phase, coexisting in a dynamic equilibrium. (B) LENS and $\tau$SOAP time-series, with the related Kernel Density Estimation (KDE) distributions, for all the water molecules (oxygen centers) in the system. Both time-series are smoothed using a moving average with width=8 ns (80 frames). Signals related to an example water molecule (ID 575) are highlighted on both LENS (red) and $\tau$SOAP (blue) components. (C) Left: Plot showing LENS and $\tau$SOAP signals related to the ID 575, while simultaneously passing from the low-intensity to the high-intensity KDE peak domain displayed in (B). Right: Projection of the whole **LEAP** dataset on the 2D LENS-$\tau$SOAP phase space (2048 water molecules x 500 frames, for a total of $\sim 1 \times 10^6$ data points). The **LEAP** path related to the ID 575 is colored from blue to yellow as time increases.

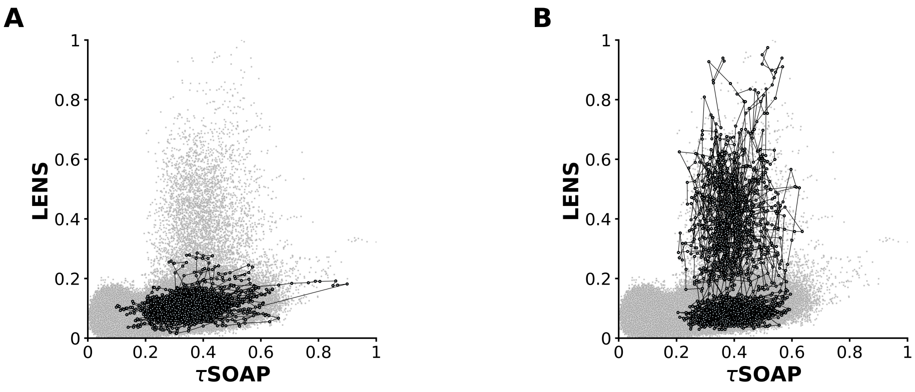


**Fig. S3. Examples of trajectory paths projected on the 2D LEAP phase space related to the Cu(211) surface at T = 600 K.** The **LEAP** dataset (related to the Cu atoms belonging to the three top-most surface layers -995 atoms- at each time step -2000 frames-, for a total of $\sim 2 \times 10^6$ data points) is projected on the 2D LENS-$\tau$SOAP phase space, displayed in gray. On such plot, two different example trajectory paths are highlighted, respectively, in (A) and (B). (A) The trajectory path related to the ID 170 is reported (black), which explores the phase space mainly moving along the $\tau$SOAP dimension. (B) The trajectory path related to the ID 59 is shown (black), which moves along the LENS dimension. Therefore, we report in (A) a first representative atom experiencing predominantly $\tau$SOAP (*structural*) fluctuations, while in (B) a second representative atom experiencing predominantly LENS (*diffusive*) fluctuations.

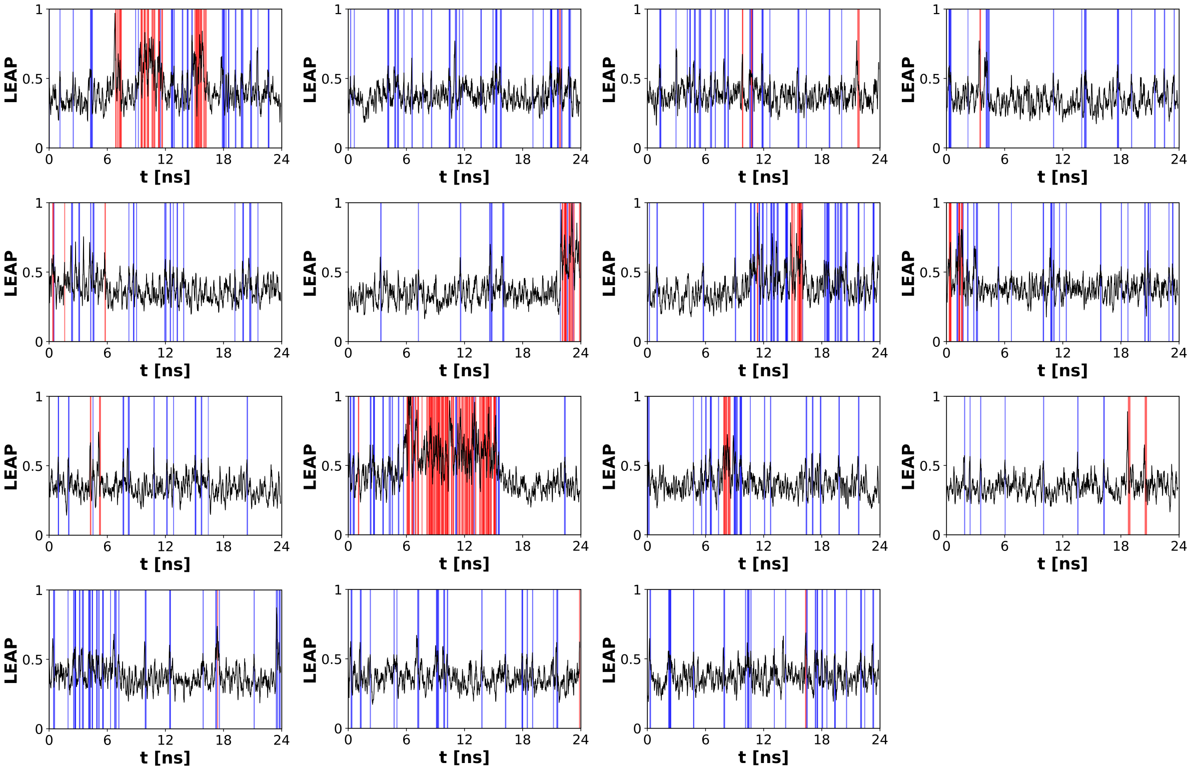


**Fig. S4. LEAP time-series of atoms experiencing diffusive fluctuations on the Cu(211) surface at T = 600 K.** Shown as black signals, **LEAP** (magnitude) time-series related to the atoms experiencing, along the trajectory, at least one predominantly LENS (*diffusive*) fluctuation. On the basis of the dynamical event they are experiencing (predominantly $\tau$SOAP or predominantly LENS), and by exploiting the classification reported in the main paper (Fig. 4), the transit through LENS and $\tau$SOAP outlier domains are identified with red and blue bands, respectively.

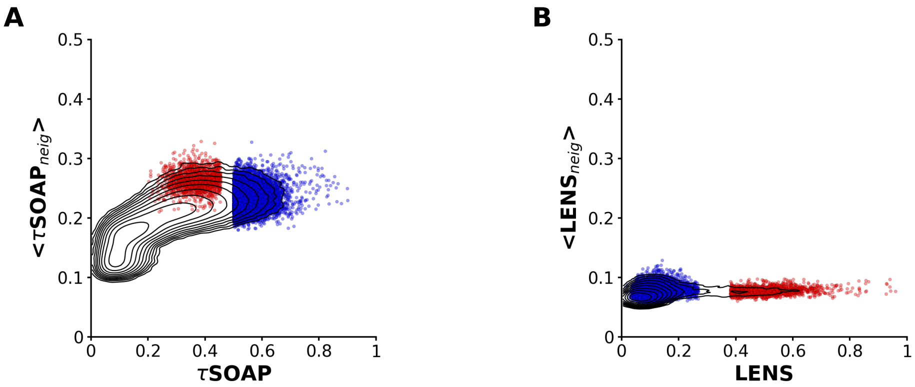


**Fig. S5. Spatial relationships in the Cu(211) surface at T=600 K.** (A) ID $\tau$SOAP value *vs.* $\tau$SOAP mean value of its neighbors. For each ID in each MD time step ($\sim 2 \times 10^6$ data points), the plot shows the relationship between the ID $\tau$SOAP value and the $\tau$SOAP mean value of its neighbors. (B) ID LENS value *vs.* LENS mean value of its neighbors. For each ID in each MD time step ($\sim 2 \times 10^6$ data points), the plot shows the relationship between the ID LENS value and the LENS mean value of its neighbors. In both plots, the red and blue points are related, respectively, to the LENS and $\tau$SOAP fluctuations identified in the main paper (Fig. 4).

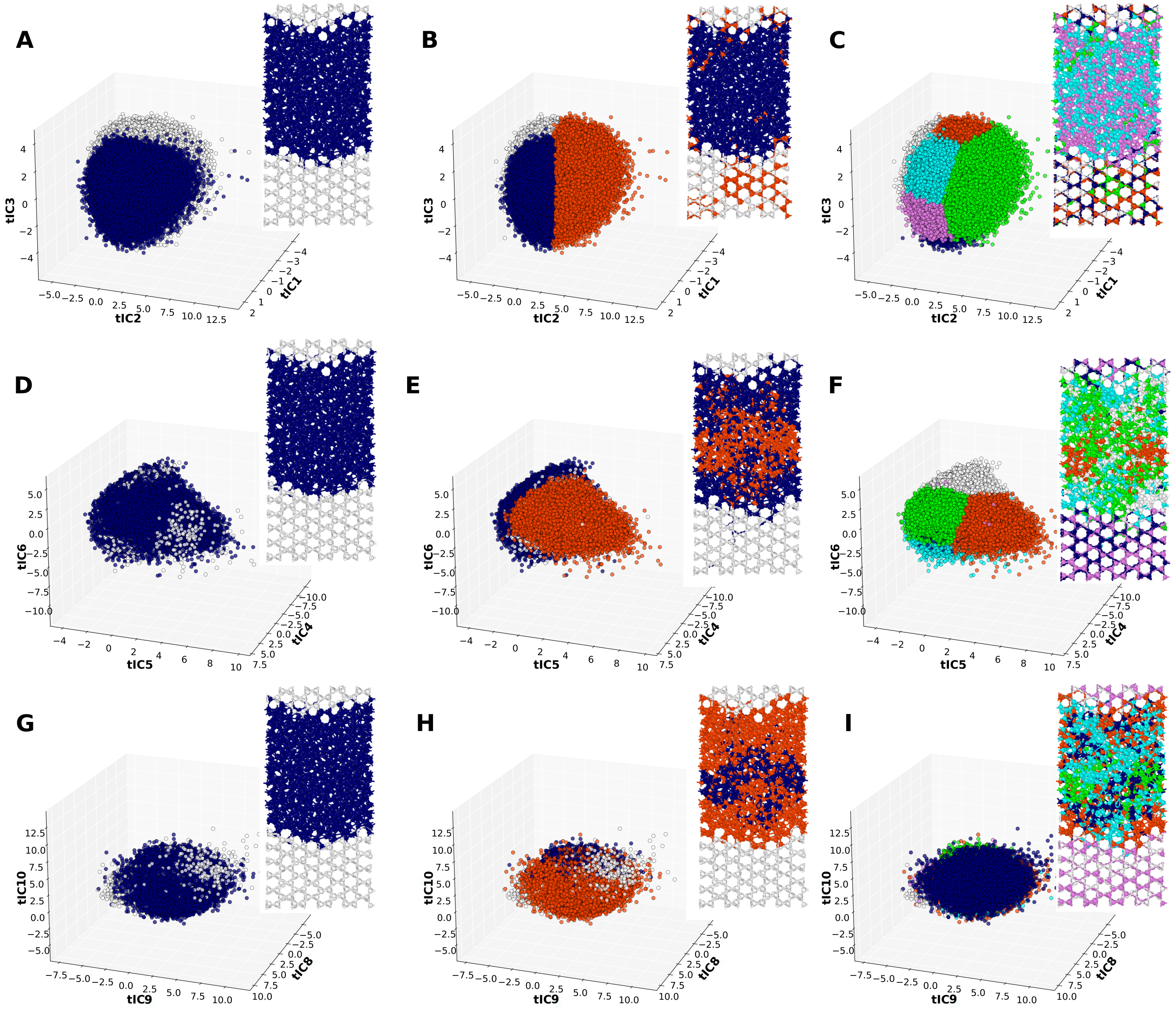


**Fig. S6.** **Time-lagged Independent Component Analysis (tICA)(4, 5) performed on the SOAP features of the ice/liquid water interface system (sampling time interval = 1 ps) and coupled to KMeans clustering. The SOAP spectra have been computed with nmax=lmax=8, rcut=6 Å considering the oxygen atoms (OW) of the water molecules as representative centers (same setup employed to get the $\tau$SOAP descriptor in the main paper, see *Materials and Methods* section). Using a lagtime=1, the tICA analysis has been performed at different *n*, that is, the kept time-lagged Independent Components (tICs), and coupled to KMeans clustering analysis. Panels A-C show the results related to the *n*=3 tIC subspace. The SOAP-based input data projected onto the first 3 tICs is reported, along with the KMeans classification of the subspace into *N*=2 (A), *N*=3 (B) and *N*=6 clusters (C ) and the corresponding snapshots. Panels D-F: *n*=6 tICs, with the last 3 tICs (tIC 4, tIC 5, and tIC 6) shown in the 3D plots. The KMeans classifications into *N*=2, 3 and 6 (with the related snapshots) are reported in D, E and F, respectively. Panels G-I: *n*=10 tICs, with the last 3 tICs (tIC 8, tIC 9 and tIC 10) reported in the 3D plots. G, H and I display, respectively, the KMeans classification of the *n*=10 tIC subspace into *N*=2, *N*=3 and *N*=6 clusters. Overall, the SOAP-based tICA analysis allows to well-distinguish the ice from the liquid water domain by keeping a low (*n*=3) as well as a higher (*n*=10) number of tICs (A, D and G). The detection of additional relevant dynamics domains, such as the ice/liquid water interface, however, is not enhanced neither by increasing the number of clusters identified by the KMeans algorithm nor by keeping a higher number of tICs.**

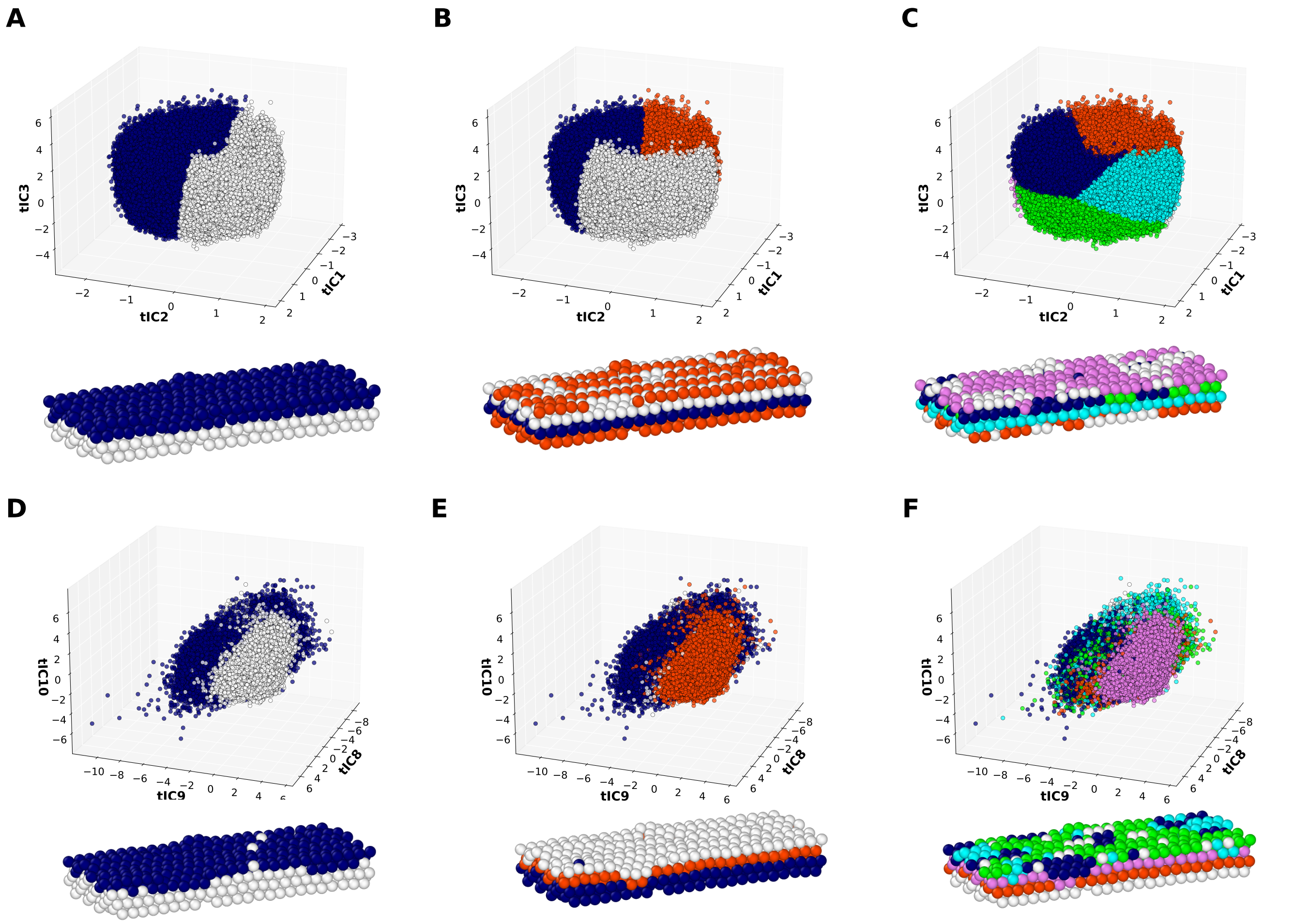


**Fig. S7. SOAP-based tICA ([4], [5]) analysis performed on the Cu(211) surface at T=600 K (sampling time interval = 12 ps) and coupled to KMeans clustering method.** The SOAP spectra have been computed with nmax=lmax=8, rcut=6 Å (same setup employed to get the $\tau$SOAP descriptor in the main paper, see *Materials and Methods* related to this system). Using a lagtime=1, the tICA analysis has been performed by keeping $n$=3 (A-C) and $n$=10 (D-F) dominant tICs. Panels A-C show the results related to the $n$=3 tIC subspace. The SOAP-based input data projected onto the first 3 tICs is reported, along with the KMeans classification of the subspace into $N$=2 (A), $N$=3 (B) and $N$=6 clusters (C ) and the corresponding snapshots. Panels D-F: $n$=10 tICs, with the last 3 tICs (tIC 8, tIC 9 and tIC 10) reported in the 3D plots. G, H and I display, respectively, the KMeans classification of the $n$=10 tIC subspace into $N$=2, $N$=3 and $N$=6 clusters. The SOAP-based tICA analysis allows to well-differentiate the bulk atoms from the surface atoms by keeping a low ($n$=3) as well as a higher ($n$=10) number of tICs. Further relevant dynamics domains, such as sliding events on the surface, cannot be isolated by means of such an approach neither by increasing the number of clusters identified by the KMeans algorithm nor by keeping a higher number of tICs.

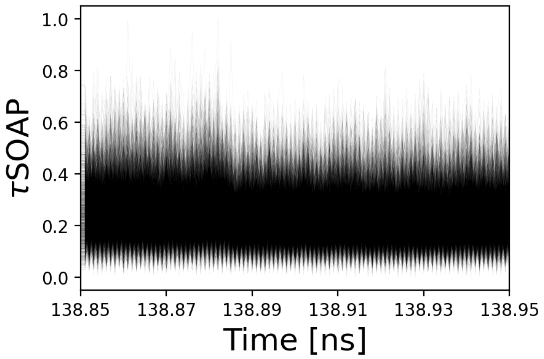


**Fig. S8.** $\tau$**SOAP component during the plastic deformation of metals.** Decomposition of the **LEAP** time-series, related to 2744 atoms in a bulk of copper (Cu) FCC crystal and subjected to a constant strain rate at $T = 300$ K, in its $\tau$SOAP component. As shown in the main paper (Fig. 5), the reference plastic event occurs at $\sim$ 130-145 ns. A zoom in the temporal evolution of the $\tau$SOAP time-series (from 138.85 ns to 138.95 ns) does not reveal any pronounced peak, differently from what observed for the LENS component at $\sim$ 138.88 ns in the main paper.

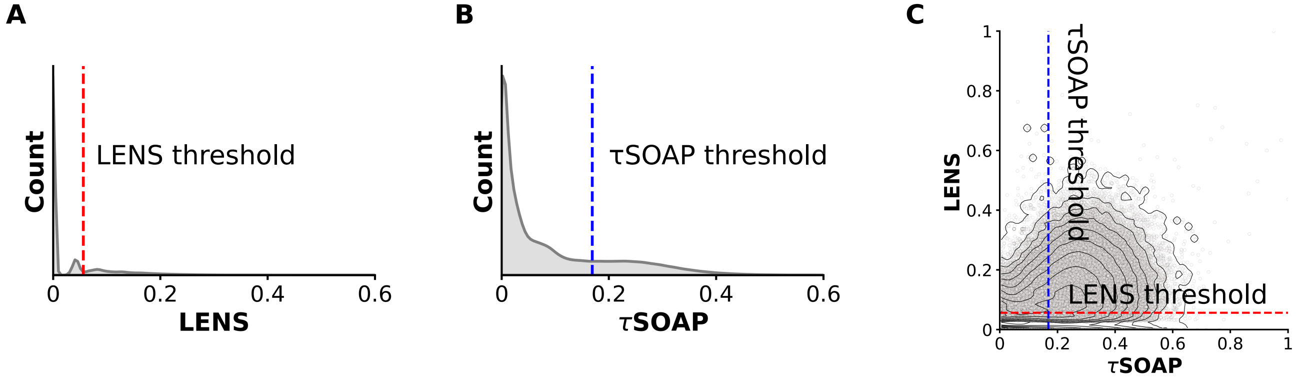


**Fig. S9. Identification of LENS and $\tau$SOAP fluctuations in the active matter system.** (A) Kernel Density Estimation (KDE) distribution of the LENS time-series computed, at each frame (200 frames in total, corresponding to 200 ms) for each particle (N = 6921). A low-density domain, characterized by fewer particles undergoing high-intensity LENS values, can be isolated by the LENS threshold (LENS = 0.056, red dashed line). The LENS threshold corresponds to the minimum value between the last two peaks identified in the KDE distribution. All the LENS values higher than the threshold are considered as LENS fluctuations. (B) KDE distribution of the $\tau$SOAP time-series computed, at each frame (200 frames in total, corresponding to 200 ms) for each particle (N = 6921). Such as in (A) for LENS, the $\tau$SOAP threshold ($\tau$SOAP = 0.169, blue dashed line) isolates a low-density domain, characterized by high-intensity $\tau$SOAP values. The $\tau$SOAP threshold corresponds to the minimum value between the last two peaks identified in the KDE distribution. All the $\tau$SOAP values higher than the threshold are considered as $\tau$SOAP fluctuations. These thresholds are used to separate fluctuations from the characteristic LENS and $\tau$SOAP vibrations (low-intensity values) in the system. (C) The thresholds identified in (A) and (B) are plotted on the projection of the **LEAP** dataset (6921 particles for 200 frames, for a total of $\sim 1.3 \times 10^6$ data points, shown in gray) on the 2D LENS-$\tau$SOAP phase space.

| SYSTEM | Trajectory Length[ns] | # of sampled frames | Sampling $\Delta t$ [ns] | Smoothing window [frames] | $r_{cut}$ | LENS-$\tau$SOAP center |
|---|---|---|---|---|---|---|
| Ice/liquid water phase coexistence | 3 | 3000 | 0.001 | 200 | 6 Å | OW |
| Cu(211) FCC 600K | 24 | 2000 | 0.012 | 10 | 6 Å | Cu |
| Cu FCC bulk 300K | 0.1 | 20 | 0.005 | - | 8 Å | Cu |
| Quincke rollers | $200 \times 10^3$ | 200 | $1 \times 10^3$ | 2 | 56 $\mu$m | C |

**Table S1. Setup details of all the LEAP analyses conducted in this work.**

**Movie S1. MD trajectory ($\sim$ 14-15 ns) of the Cu(211) FCC surface used for the analyses of Fig. 3 and Fig. 4 in the main text, colored according to the classification reported in Fig. 4B. Sliding Cu atoms, related to the LENS-dominant fluctuations region, can be observed in red; Cu atoms undergoing structural rearrangements, related to the $\tau$SOAP-dominant fluctuations region, are shown in blue; all the other Cu atoms, which are not undergoing relevant fluctuations, are shown in white.**

**Movie S2. Trajectory (0-145 ms) obtained from the experimental movie of dielectric colloidal particles used for the analyses of Fig. 6 in the main text, colored according to the classification shown in Fig. 6C. Four domains can be identified: in white, the quiescent particles; in lilac, the core of the wave; in red, units undergoing LENS-dominant fluctuations, mainly corresponding to the wavefront; in blue, units undergoing $\tau$SOAP-dominant fluctuations.**

## References


1. M Becchi, F Fantolino, GM Pavan, Layer-by-layer unsupervised clustering of statistically relevant fluctuations in noisy time-series data of complex dynamical systems. *Proc. Natl. Acad. Sci. U.S.A.* **121**, e2403771121 (2024).
2. J Abascal, E Sanz, R García Fernández, C Vega, A potential model for the study of ices and amorphous water: Tip4p/ice. *J. Chem. Phys.* **122**, 234511 (2005).
3. R García Fernández, JL Abascal, C Vega, The melting point of ice ih for common water models calculated from direct coexistence of the solid-liquid interface. *J. Chem. Phys.* **124**, 144506 (2006).
4. L Molgedey, HG Schuster, Separation of a mixture of independent signals using time delayed correlations. *Phys. review letters* **72**, 3634 (1994).
5. G Pérez-Hernández, F Paul, T Giorgino, G De Fabritiis, F Noé, Identification of slow molecular order parameters for markov model construction. *The J. chemical physics* **139** (2013).
